# E2F1 Hinders Skin Wound Healing by Repressing Vascular Endothelial Growth Factor (VEGF) Expression, Neovascularization, and Macrophage Recruitment

**DOI:** 10.1371/journal.pone.0160411

**Published:** 2016-08-04

**Authors:** Ningning Wang, Yiping Wu, Ning Zeng, Haiping Wang, Pei Deng, Yi Xu, Youping Feng, Hong Zeng, Hongxia Yang, Kai Hou, Andrew Wang, Keshav Parthasarathy, Samaksh Goyal, Gangjian Qin, Min Wu

**Affiliations:** 1 Department of Plastic Surgery, Tongji Hospital, Tongji Medical College, Huazhong University of Science and Technology, Wuhan, Hubei, China; 2 College of Liberal Arts and Science, University of Illinois Urbana-Champaign, Illinois, United States of America; 3 Feinberg Cardiovascular Research Institute, Department of Medicine – Cardiology, Northwestern University Feinberg School of Medicine, Chicago, Illinois, United States of America; 4 Department of Biomedical Engineering, the University of Alabama at Birmingham, School of Medicine and School of Engineering, Birmingham, Alabama, United States of America; University of Illinois at Chicago, UNITED STATES

## Abstract

**Background:**

Refractory surface of wound and dermal chronic ulcer are largely attributed to poor neovascularization. We have previously shown that E2F1 suppresses VEGF expression in the ischemic heart, and that genetic deletion of E2F1 leads to better cardiac recovery. However, whether E2F1 has a role in dermal wound healing is currently not known.

**Methods and Results:**

Skin wounds were surgically induced in E2F1-null (E2F1^–/–^) mice and WT littermates. E2F1^–/–^ displayed an accelerated wound healing including wound closure, dermal thickening and collagen deposition, which was associated with an increased endothelial cell proliferation and greater vessel density in the border zone of the wound. Furthermore, more macrophages were recruited to the skin lesions and the level of VEGF expression was markedly higher in E2F1^–/–^ than in WT mice.

**Conclusions:**

E2F1 hinders skin wound healing by suppressing VEGF expression, neovascularization, and macrophage recruitment. Strategies that target E2F1 may enhance wound healing.

## Introduction

The wound caused by trauma, diabetes mellitus or surgery affects the patients’ quality of life [1]. The healing process is characterized typically by interactions of a complex cascade of cellular events, including inflammatory cell (especially macrophage) infiltration, dermal thickening (tissue growth) and collagen deposition (tissue maturation) [2, 3]. It has been recognized that the insufficiency of new blood vessel formation (neovascularization) is an important factor affecting the repair and regeneration of injured tissue [4–6]. We have previously reported that in a mouse model of myocardial infarction, an increase in neovascularization can significantly reduce infarct size [7]. Others have also shown that anti-vascular tumor therapies are often associated with wound repair deficiencies [8]. In contrast, enhancing vascular formation can shorten the time of wound healing in diabetic animals [9].

E2F1 is a transcription factor that regulates cell growth and survival [10]. However, accumulating evidence indicates its diverse physiological functions beyond cell cycle regulation [11]. Previously, we have reported that E2F1 is involved in the regulation of cardiac angiogenesis; genetic deletion of E2F1 in mice enhances blood flow recovery after ischemic injury [12]. More recently, we found that E2F1 binds to VEGF promoter and repress VEGF transcription in response to hypoxia [13]. However, whether E2F1 is involved in the regulation of wound healing is currently not known. Here we found that E2F1 is a repressor of skin wound healing, and that loss of E2F1 accelerates wound healing by increasing VEGF expression and macrophage recruitment.

## Methods

### Mice

The heterozygote E2F1^+/–^ mice were obtained from Jaxson laboratory (http://jaxmice.jax.org/strain/002785.html) and were bred, maintained, and operated in the Animal Experimental Center of Tongji Hospital, Huazhong University of Science and Technology. All animals were genotyped by PCR of tail DNA following the Jax lab protocol. The experiments were approved by the Ethical Committee of the Tongji Hospital, Tongji Medical College at the Huazhong University of Science and Technology in China (IRB ID: TJ-A20121213). Mice were euthanized by carbon dioxide (CO2) inhalation, followed by cervical dislocation as the second physical means to ensure death.

### Surgical induction of skin wound healing model

Skin wound healing model was induced in male E2F1^–/–^ mice and their WT littermates at 10–12 weeks of age as we described previously [14]. Mice were anesthetized with Isoflurane delivered at 2–4% during the surgery, then the dorsal surface was shaved, washed with povidone-iodine solution and alcohol, and a 0.9cm punch biospy tool was used to create one full-thickness excisional skin wound. After surgery, Buprenex (1–2 mg/kg, *i*.*p*.) was administered every 12 h and continued for 3 days to reduce pain.

### Histological assessments of wound size and collagen deposition

Wounds were photographed with a camera (Canon PowerShot A640), and the images were analyzed by calculating the enclosed pixel area with the NIH Image J software. The percentage of wound closure was calculated by using the following equation: Wound closure (%) = (Wound area on day 0 –Wound area on the indicated day) / Wound area on day 0 x 100% [14]. Collagen contents in paraffin-embedded skin sections were determined by using quantitative micro-assay kit (Chondrex, Inc, Redmond, WA) following the manufacturer’s instructions [15]. This method is based on the selective binding of Sirius Red and Fast Green to collagens and non-collagen proteins. Briefly, 10 mm-thick sections were deparaffinized, and stained with Sirius Red and Fast Green. The color in the tissue sections was eluted by dye extraction solution. Absorbance was measured in a spectrophotometer at OD540 (for Sirius Red) and OD605 (for Fast Green), respectively. The relative collagen amount is calculated as the ratio of OD values of collagens to non-collagen proteins.

### HE and Masson’s trichrome staining

Skin samples were paraffin-embedded, and sections were dewaxed in xylene and rehydrated in graded alcohols. HE staining or Masson’s trichrome staining was performed as described [16].

### Histological assessments of vessel density and proliferating endothelial cells (ECs)

Skin tissues were analyzed for vascular density (Lectin I staining) and cell proliferation (5-bromodeoxyuridine [BrdU] staining) as we described previously [13, 17]. Positive cells were counted in five random fields. Data were expressed as the mean ± SEM of six independent counts.

### Quantitative RT-PCR

The mRNA expression in the wounded skin tissues was evaluated with qRT-PCR as we previously reported, with primers for murine VEGF-A [13] and M1 and M2 macrophage markers [18].

### Western blotting

Western blotting was performed with 30 μg of whole-cell lysate by following standard techniques, as we previously described [13]. The primary antibodies used were anti-VEGF and anti-tubulin (Santa Cruz Biotechnology, Inc, Santa Cruz, CA). Band intensities were determined densitometrically with Image J software, and the relative level of VEGF was normalized to tubulin levels.

### Statistical analysis

All values were expressed as mean ± SEM. Comparison between two means was performed with an unpaired Student’s t test, whereas ANOVA with Fisher’s protected least significant differences and Bonferroni—Dunn post hoc analysis were used for comparisons of more than two means. Significance was defined as P<0.05.

## Results

### E2F1 deficient (E2F1^–/–^) mice display an accelerated wound healing after skin injury

**Fig 1:**
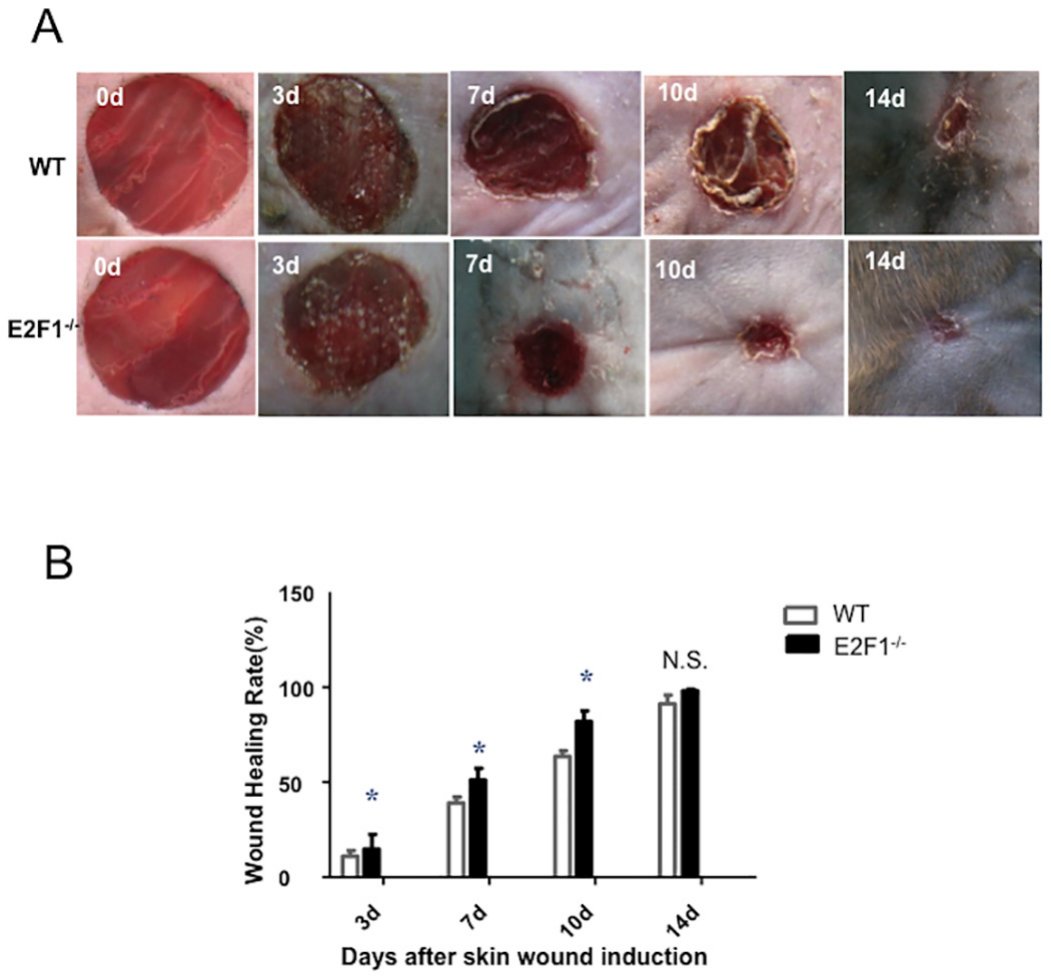
E2F1-deficiency accelerates wound healing. (A) Representative skin wounds in WT and E2F1^–/–^ mice at serial time points post- surgery. (B) Quantifications of the wound healing rates (percent wound closure). *p<0.05 vs. WT at same time points. N.S., not significant; n = 10 per group.

We induced skin wound in E2F1^–/–^ mice and control wild-type (WT) littermates. The wound sizes were measured at 0, 7, 10 and 14 days after the surgical procedure. E2F1^–/–^ mice exhibited a significant smaller wound area, thus a greater percent wound closure at days 3, 7 and 10 as compared to WT mice (Fig 1A and 1B).

### The dermal thickness and collagen amount are increased in the skin wound of E2F1^–/–^ mice

**Fig 2:**
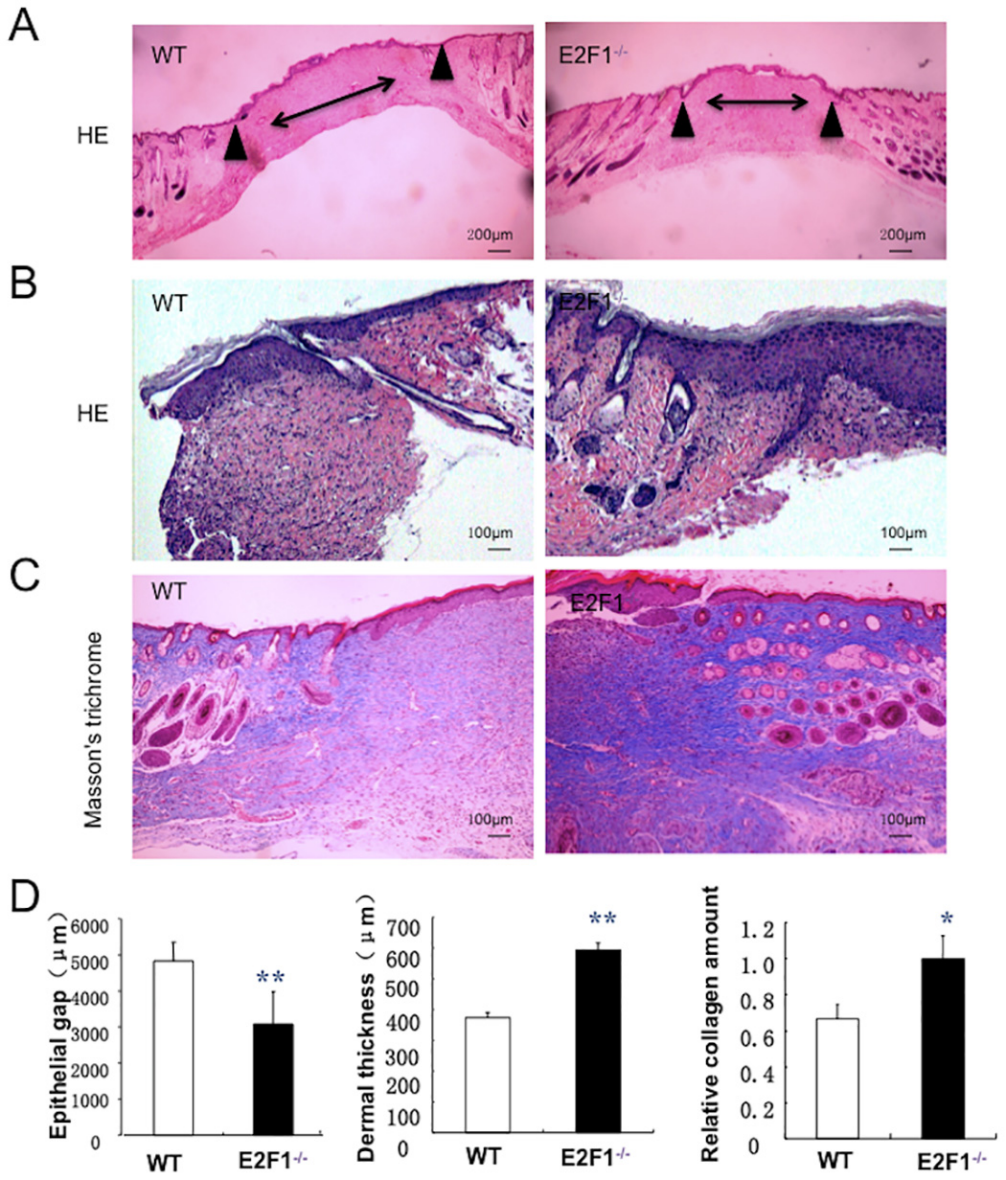
The dermal thickness and collagen amount are greater in E2F1^–/–^ mice than in WT mice. Wound tissues were isolated at day 7 post-surgery and analyzed histologically. (A-B) H.E. and (C) Masson’s trichrome staining. Both entire wound (A) and wound border zone (B-C) are shown. Arrowheads indicate edges of wound granulation tissue. Double-headed arrow bars indicate the distance between the leading edges of wounded epidermis. (D) Quantifications of Epithelial gap (*left panel*), dermal thickness (*middle panel*), and collagen deposition (*right panel*). **p<0.01, *p<0.05; n = 10 per group.

We harvested the wound skin in the WT and E2F1^–/–^ mice at day 7 post-surgery. H & E staining confirmed the smaller wound size in E2F1^–/–^ mice (Fig 2A). Masson’s trichrome staining and immunohistochemical analyses revealed that in the wound border zone, E2F1^–/–^ mice had thicker dermis and more collagen deposition than WT controls (Fig 2B–2D).

### E2F1^–/–^ mice exhibit an enhanced neovascularization in the border zone of the skin wound

**Fig 3:**
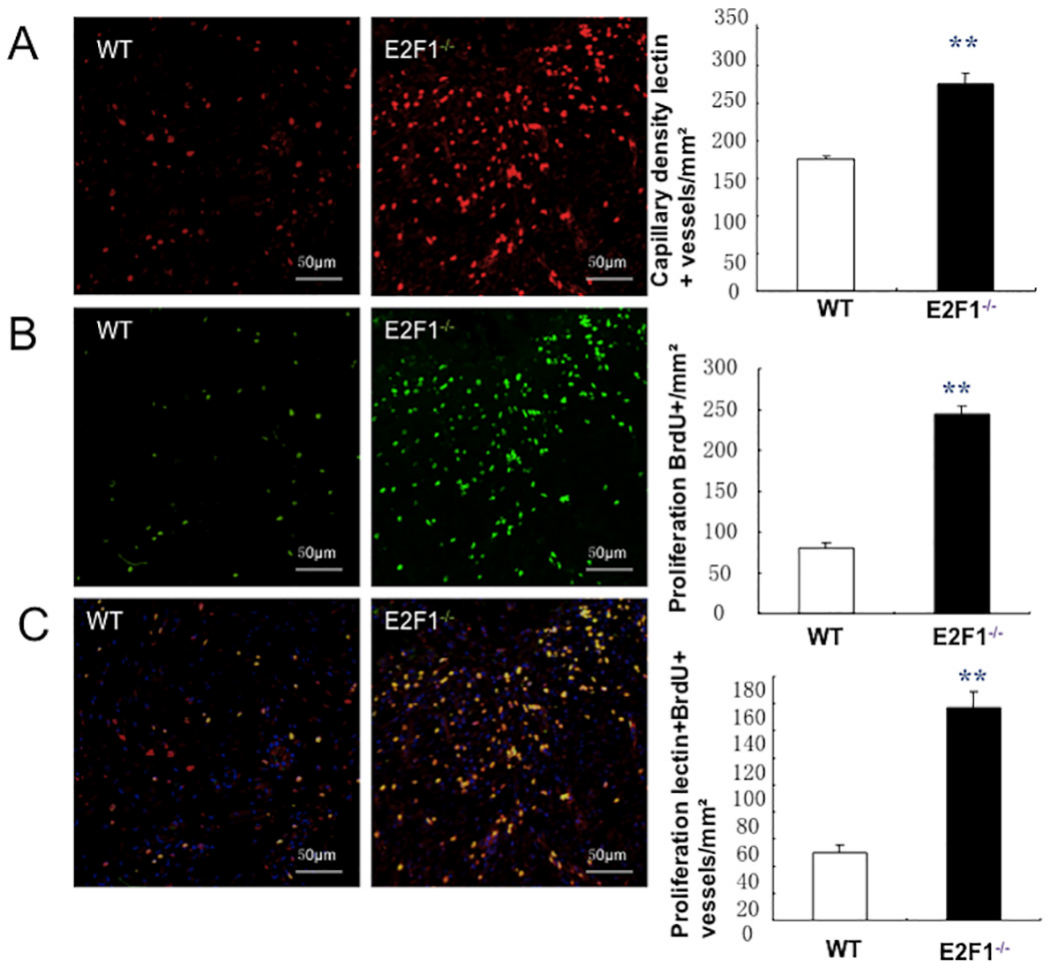
E2F1-deficiency enhances vessel growth in the border area of the skin wound. At day 7 post-surgery, Lectin was i.v. injected 10 min before euthanasia to identify vasculature; subsequent analyses were performed in sections of wound skin and quantified at the border zone of wounds. (A) Functional vessels were identified by staining with anti-lectin antibodies (red) (*left and middle panels*, scale bar = 50mm) and quantified (*right panel*). (B) Proliferation cells were identified by staining for BrdU (green) (*left and middle panels*) and quantified (*right panel*). (C) Proliferating ECs (yellow) were identified by co-staining for BrdU (green) and lectin (red) (*left and middle panels*, scale bar = 50 mm) and quantified as the number of lectin+BrdU+ vessels per mm^2^ (*right panel*); nuclei were stained with DAPI (blue). **p<0.01; n = 10 per group.

On 7^th^ day after skin wound or sham operation, the functional vasculature was labeled by i.v. injection of lectin before euthanasia; then capillary density and EC proliferation were evaluated by staining for lectin and co-staining for lectin and BrdU, respectively. Notably, in the border area of the wound skin, capillary density is significantly greater and proliferation ECs were significantly more abundant in E2F1^–/–^ mice than in WT mice (Fig 3).

### E2F1^–/–^ mice have more macrophages in the wounded skin

**Fig 4:**
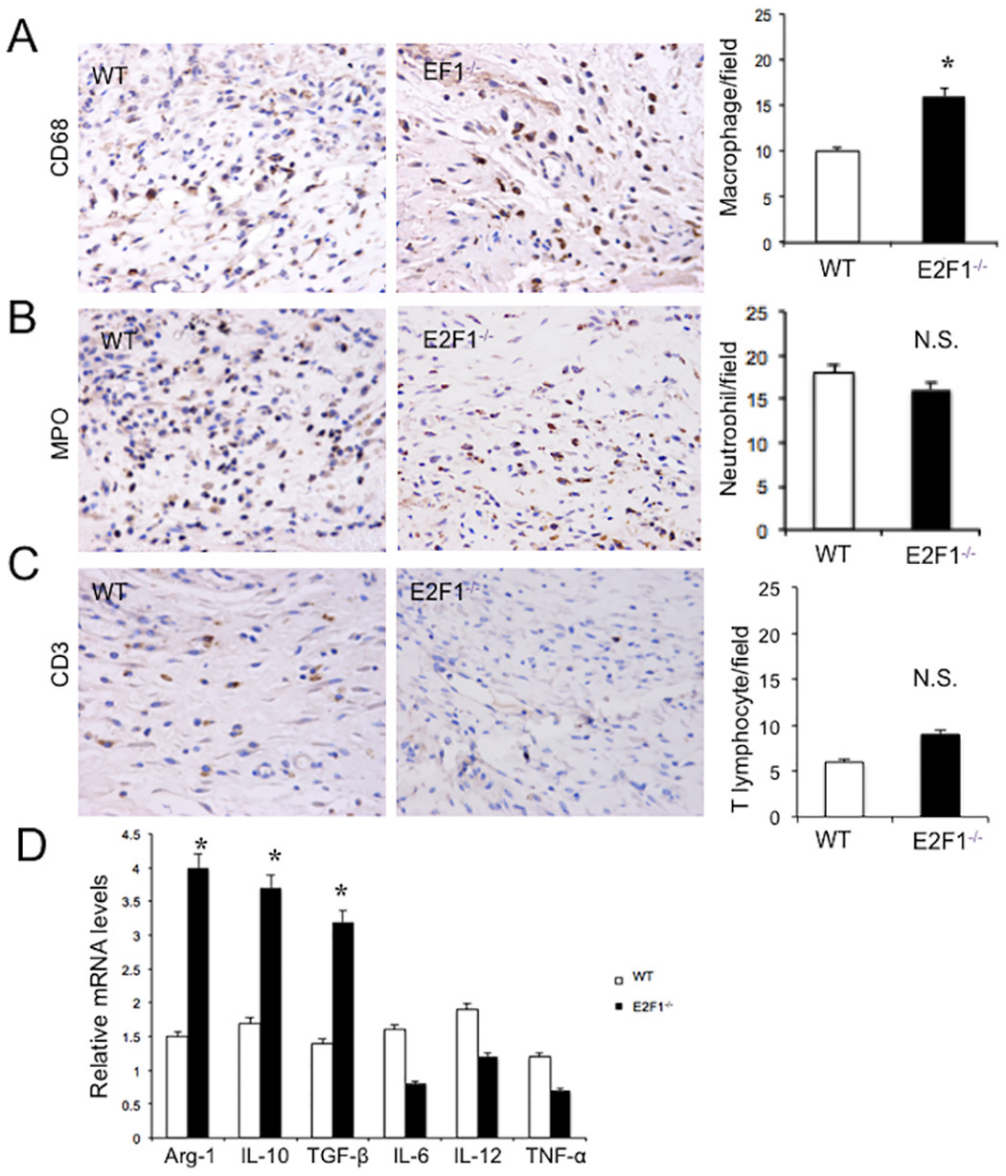
More infiltrating macrophages are found in the wound of E2F1^–/–^ mice than in WT mice. At day 7 post-surgery, the wound skin tissues were analyzed immunochemically and by qRT-PCR. Shown are representative staining (*left and middle panels*) (original magnification, X400; 0.06 mm^2^/field) and quantifications (*right panels*) of the cells stained positive for CD68 (A), MPO (B) and CD3 (C). (D) The expression levels of M1 and M2 macrophage marker genes in the wounded skin tissues of WT and E2F1^–/–^ mice were analyzed by qRT-PCR. **p<0.01. n.s., not significant; n = 10 per group.

To understand the mechanism by which E2F1-deficiency enhances cutaneous fibrosis, we compared the number of inflammatory cells between E2F1^–/–^ and WT mice by immunohistochemical staining for CD68, MPO, and CD3 at 7 day after surgery. In the border zone of skin wounds, the number of macrophages (CD68+) in E2F1^–/–^ mice was significantly more than in WT mice (Fig 4A). However, no difference was seen in the numbers of neutrophils (MPO+) and T cells (CD3+) (Fig 4B and 4C) between the two groups of mice. Importantly, the expression levels of M1 and M2 macrophage marker genes in the wounded skin tissues were analyzed by qRT-PCR. The levels of M2 markers, including Arg-1, IL-10, and TGF-β were significantly higher in E2F1^–/–^ mice than in WT mice, which is compatible with a pro-angiogenic and wound healing phenotype.

### Loss of E2F1 leads to increased VEGF expression in the skin wound

**Fig 5:**
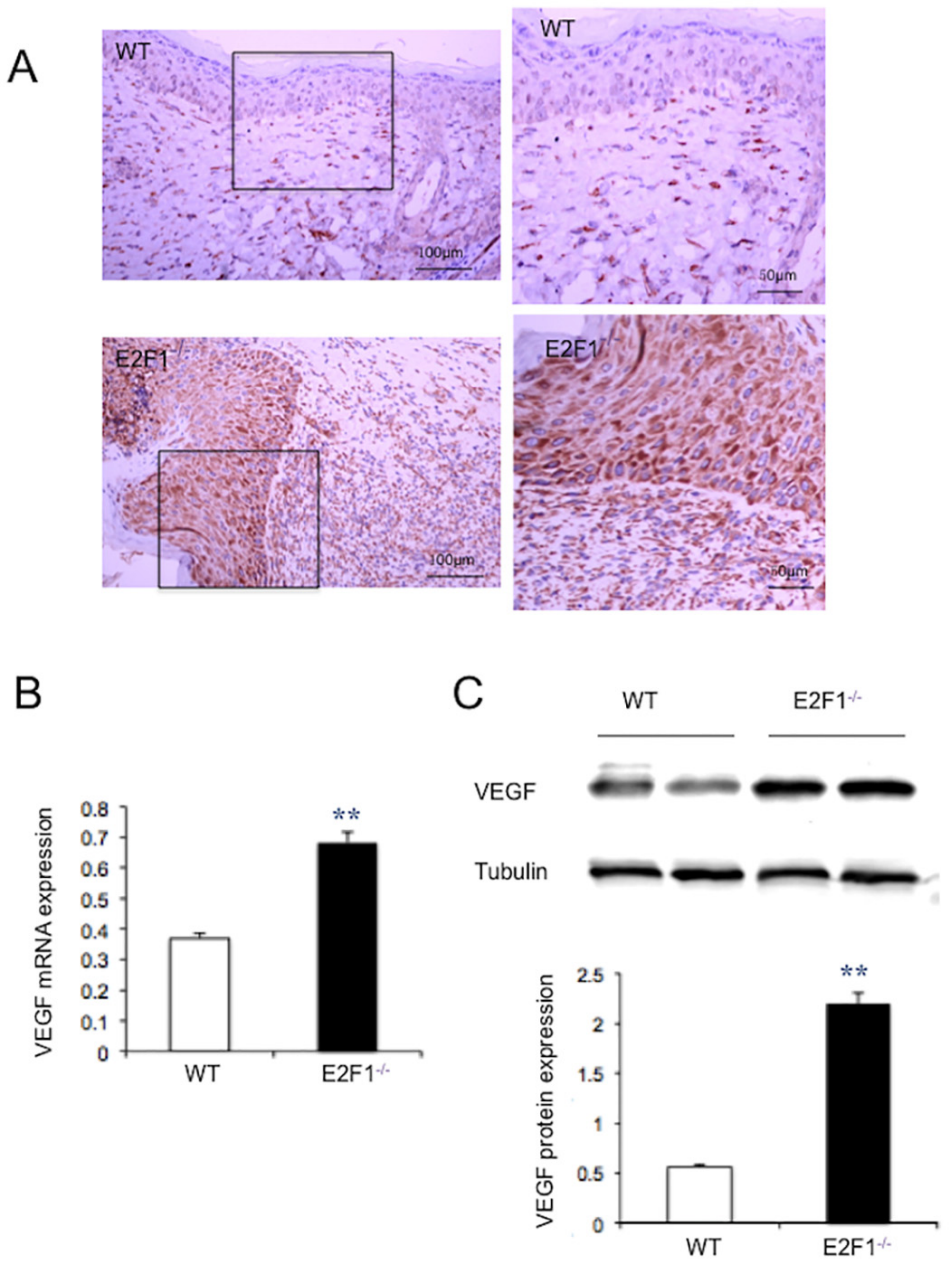
VEGF-A level is significantly higher in the wound of E2F1^–/–^ mice than in WT mice. Wound tissues were isolated at day 7 post-surgery. (A) Immumohistochemical staining of VEGF (brown), 20X original magnification (*left panels*), the inlets were enlarged (*right panels*). (B) VEGF mRNA expression was analyzed by qRT-PCR and normalized to the level of GAPDH. (C) Representatives of Western blotting (*upper panel*) and quantification of VEGF protein levels (*lower panel*). Protein levels were quantified densitometrically and normalized to tubulin levels. **p<0.01; n = 5.

We previously reported that E2F1 binds to the VEGF promoter in the ischemia myocardium [13]. To understand the role of VEGF in the enhanced EC growth in the skin wounds of E2F1^–/–^ mice, we performed qRT-PCR, immunohistochemical staining and Western blotting to detect VEGF expression in the tissue at day 7 post-surgery. In the border zone, the levels of VEGF mRNA and protein were significantly higher in E2F1^–/–^ mice than in WT mice (Fig 5). Thus, induction of VEGF expression by skin wounding appears to be repressed by endogenous E2F1.

## Discussion

In this study we disclose that genetic deletion of E2F1 enhances skin wound repair by upregulating VEGF expression and increasing macrophage recruitment. Wound healing is a complex process involving different cell types: inflammatory cells, fibroblasts, keratinocytes and ECs [3, 19]. These cells release various chemo-cytokines and growth factors to initiate inflammation, new blood vessel formation, and tissue remodeling. In the angiogenesis phase, the new vessel density determines the rate of the healing process [4]. In the current study, we found that E2F1-deficiency increase skin wound healing rate via new vessel formation. The newly formed vessels not only allow leukocyte migration into the wound, but also supply the oxygen and nutrients necessary to sustain the growth of granulation tissues.

VEGF is perhaps the most important vascular growth factor that is regulated by hypoxia. Over past years, much new information on the signals controlling wound cell behavior has emerged, which has led to a number of novel therapeutic strategies including VEGF administration [20]. However, the clinical trials have achieved rather limited success, at least in part, due to the difficulty in the delivery to injured tissues and the short half-life of the exogenous VEGF protein [21]. Our results suggest that inhibition of endogenous E2F1 may serve as an alternative approach to ensure continuous expression of VEGF in the wound and this is particularly useful for certain pathological conditions (e.g., diabetes), in which hypoxic response and VEGF induction are impaired [22].

E2F1 is classically considered an ‘activating’ transcription factor, because its overexpression transactivates target genes, leading to cell-cycle progression [10]. However, recent evidence from our lab and others’ suggest that E2F1 loss of function protects ischemic tissue injury [12, 13, 23]. In particular, we have shown that E2F1 is a critical repressor of VEGF expression and new vessel formation. The findings in the current report are consistent with our previous observations and further extend the physiological significance of this important regulatory pathway to the physiology of cutaneous wound healing.

In addition, we found more macrophages in the wound skin of E2F1^–/–^ mice than in WT mice. This is exciting because the role of macrophages in skin wound healing has been increasingly recognized [24]. Although the detailed mechanism is still not known, it is likely that the elevated VEGF expression plays a role since VEGF receptor 1 is also expressed in monocytes/macrophages [25]. Interestingly, previous studies have showed that transplantation of EPCs accelerates dermal wound healing with increased recruitment of monocytes/ macrophages [26], and that mobilization of human CD34+ hematopoietic stem cells from bone marrow to the peripheral blood is associated with E2F1 downregulation [27]. Thus an alternative explanation for more macrophages in the E2F1^–/–^ mice might be deletion of E2F1 accelerating EPC mobilization to the wound. Nevertheless, further study is required to understand the precise mechanisms underlying E2F1 regulation of macrophage recruitment.

The rate of wound healing was significantly different between the E2F1^–/–^ mice and WT mice at early time points (days 3, 7 and 10), but not at later time point (day 14). This suggests that VEGF expression and angiogenesis may not be the only contributor to the normal wound healing process. However, it is conceivable that under certain pathological conditions in which wound healing is limited by impairment of angiogenesis, an enhanced angiogenesis may allow wound recovery to a level unmatched. We will consider testing this hypothesis by testing the effect of E2F1 deficiency in diabetic mouse models in our future study.

In conclusion, our data suggest that E2F1 hinders skin wound healing by suppressing VEGF expression, neovascularization, and macrophage recruitment. Thus strategies that target E2F1 may enhance wound healing.
